# 

**Published:** 1823-06-01

**Authors:** 


### Contents

OF

No. 13, Vol. IV. for JUNE 1823.

Art. I.

SUICIDE.

1. Esquirol on Suicide - - - - - - - - - - - - - - } 1

2. Falret on Suicide - - - - - - - - - - - - - - }

II.

Mr. Ellis's Biography of Dr. Gordon - - - - - - - - 29

III.

PHYSIOLOGY OF THE CIRCULATION.

1. Dr. Lucas's Principles of Inflammation - - - - - - } 38
2. Dr. Parry on the Pulse - - - - - - - - - - - - }

IV.

Dr. Chapman's Elements of Therapeutics and Materia Medica 61

V.

DUBLIN HOSPITAL REPORTS, VOL. III.

Art. 1. Mr. Todd on Aneurism - - - - - - - - - - - - 77
2. Dr. Douglas on Puerperal Fever - - - - - - - - 83
3. Dr. Speer on the Diseases of Dublin - - - - - - 88
4. Dr. Colles on Dissection Wounds - - - - - - - 95

(Reports to be continued.)

VI.

Sir Alex. Crichton on Pulmonary Consumption - - - - - 102

VII.

Dr. Cooke on Epilepsy - - - - - - - - - - - - - - 119

VIII.

Mr. Wilkinson on Cutaneous Diseases - - - - - - - - 132

IX.

DYSENTERY.

1. Dr. Cheyne on Dysentery - - - - - - - - - - - } 136

2. Dr. O'Brien on Dysentery - - - - - - - - - - - }

X.

Dr. Good's Study of Medicine - - - - - - - - - - 155

XI.

Professor SCARPA on Scirrhus and Cancer. By Mr. BRIGGS. - 172

XII.

QUARTERLY PERISCOPE.

1. Dr. Scott on Dracunculus - - - - - - - - - - - - 182
2. Mr. Brodie on the Bile - - - - - - - - - - - - - 186
3. Mr. Shaw on Laryngeal Disease - - - - - - - - 188
4. Dr. Hume on Phthisis - - - - - - - - - - - - - 189
5. M. Dupuytren on Prolapsus Ani - - - - - - - - 191
6. Mr. Earle on Ununited Fractures - - - - - - - - 192
7. Mr. Austin on Bronchocele - - - - - - - - - - - 193
8. Mr. Wade on Enteritis - - - - - - - - - - - - - *ib.*
9. Sir Astley Cooper on dilatation of the Female Urethra - *ib.*
10. Mr. Charles Bell on Cancer Mammæ - - - - - - - 195
11. Dr. Heineken on Diabetes Mellitus - - - - - - - - 200
12. Mr. B. Hutchinson on Tetanus - - - - - - - - - - *ib.*
13. Drs. Christison and Coindet on Oxalic Acid - - - - 201
14. Mr. Gilder on Vaccine and Measles - - - - - - - - 205
15. Remedy for Ascarides - - - - - - - - - - - - - 206
16. Mr. Pretty on Petechiæ Hæmorrhagicæ - - - - - - *ib.*
17. Mr. Vincent's Operation on the Neck - - - - - - - 207
18. Acetate of Morphia - - - - - - - - - - - - - - 208
19. Remarkable Abdominal Disease - - - - - - - - - 210
20. Dr. Goodisson on the Ionian Isles - - - - - - - - 211
21. Bronchotomy - - - - - - - - - - - - - - - - - 213
22. Small-Pox and Varicella - - - - - - - - - - - - 214
23. Rheumatic Metastasis - - - - - - - - - - - - - 215
24. Mr. Broughton on Cubebs - - - - - - - - - - - - 216
25. The Knife Eater - - - - - - - - - - - - - - - - 218
26. Abdominal Tumour - - - - - - - - - - - - - - - 220
27. Mr. Edwards's Case of Poisoning - - - - - - - - 221
28. Mercury in Acute Diseases - - - - - - - - - - - 222
29. Dr. Akenside on Cancer - - - - - - - - - - - - - *ib.*
30. Mr. Dowler on the Products of Inflammation - - - - 223
31. Sir Astley Cooper on Dislocations and Fractures—Fracture of the Cervix Femoris - - - - - - - - - 225

XIII.

Bibliographical Record - - - - - - - - - - - - - 229

XIV.

EXTRA LIMITES.

1. Mr. LIZARS' Reply to a Critique in the Medico-Chirurgical Review - - - - - - - - - - - - - 232

2. Mr. LOWE's Letter to Mr. Lizars - - - - - - - - - *ib.*

Intelligence, Correspondence, &c. - - - - - - - - - 234
To Correspondents - - - - - - - - - - - - - - - 239
Additional Subscribers - - - - - - - - - - - - - 240

